# Impact of enzyme replacement therapy on survival in adults with Pompe disease

**DOI:** 10.1186/1471-2474-14-S2-P15

**Published:** 2013-05-29

**Authors:** Deniz Güngör, Michelle Kruijshaar, Iris Plug, Ralph D’Agostino, Marloes Hagemans, Arnold Reuser, Ans van der Ploeg

**Affiliations:** 1 Department of Pediatrics, Center for Lysosomal and Metabolic Diseases, Erasmus MC University Medical Center, Rotterdam, The Netherlands; 2 Department of Clinical Genetics, Center for Lysosomal and Metabolic Diseases, Erasmus MC University Medical Center, Rotterdam, The Netherlands; 3 Department of Mathematics and Statistics, Boston University, Harvard Clinical Research Institute, Boston, MA, USA

## Background

Since 2006, enzyme replacement therapy (ERT) has been available as a treatment for patients with Pompe disease. ERT has shown efficacy concerning muscle strength and pulmonary function in adult patients. However, no data on the effect of ERT on the survival of adult patients are currently available. Our objective was to assess the effect of ERT on survival in adult patients with Pompe disease.

## Methods

Data were collected as part of an international observational study conducted between 2002 and 2011 in which patients were followed on an annual basis. Time dependent covariate Cox’s proportional hazards models were used for univariate and multivariate analyses of the risk of death. Patients who discontinued treatment were censored at the time of discontinuation. Additionally, we used an ‘intention to treat’ approach.

## Results

Overall, 283 adult patients with a median age of 48 years (range, 19-81 years) were included in the study. Seventy-two percent of the patients started ERT at some time during follow-up and 28% never received ERT. During follow-up (median, 6 years; range, 0.04 to 9 years), 46 patients died, 28 (61%) of whom had never received ERT. After adjustment for age, gender, country of residence, and disease severity (based on wheelchair and ventilator use), ERT was positively associated with survival (hazard ratio 0.41, CI 95 % 0.19-0.87). The hazard ratio for ERT in the multivariable analyses of the intention to treat approach was 0.33 (CI 95 % 0.15-0.73).

## Conclusion

Our prospective study provides novel data on the positive effect that ERT has on survival in adults with Pompe disease. Given the fact that ERT was only registered in 2006, this may be considered as a very promising finding.

